# Management of high cows-share-contribution of SCC to the bulk milk tank by acoustic pulse technology (APT)

**DOI:** 10.1371/journal.pone.0255747

**Published:** 2021-08-23

**Authors:** Uzi Merin, Gabriel Leitner, Shamay Jacoby, Dani Gilad

**Affiliations:** 1 Emeritus Senior Scientist, Department of Food Quality and Safety, Institute of Postharvest and Food Sciences, The Volcani Center, Bet Dagan, Israel; 2 Armenta Ltd., Ra’anana, Israel; 3 Institute of Animal Science, A.R.O., The Volcani Center, Bet Dagan, Israel; Tokat Gaziosmanpasa Universitesi, TURKEY

## Abstract

A cow with mastitis has a high somatic cell count (SCC) in its milk. Cow-share-contribution of somatic cells to the bulk milk tank (BMTSCC) refers to the relative addition made by each cow’s milk to the bulk tank’s SCC. Since bulk milk is graded and priced according to the BMTSCC, high-yielding cows with mastitis are the main contributors to penalizations in milk price. The benefits of acoustic pulse technology (APT) application to tissues are well documented, including its anti-inflammatory effect and restoration of tissue function by triggering natural healing processes. An APT-based device was developed specifically for treating mastitis in dairy cows. It enables rapid and deep penetration of the acoustic pulses over a large area of the udder in a single session. A study was performed on six farms with a total of 3,900 cows. One unit of cow-share-contribution equaled the addition of 1,000 cells to each mL of the bulk milk volume above the mean BMTSCC. A total of 206 cows were selected: 103 were treated with APT and 103 served as controls. All of the cows contributed over 1.5 units to the BMTSCC at the time of treatment. Seventy-five days after APT treatment, 2 of the 103 treated cows (1.9%) were culled, compared to 19 (18.5%) of the 103 control cows, as well as infected quarter dry-off in 5 others (4.85%). Overall success was defined as a decrease of >75% in cow-share-contribution from treatment time in two of the three monthly milk recordings following treatment. Results indicated 57.3% success for the APT-treated cows vs. 14.6% for the untreated control groups. Highest share-contribution provide an additional tool for the farmer’s decision of how to control BMTSCC. Because the cow-share-contribution value is relative to herd size and BMTSCC, this study included a similar number of cows, with similar SCC and milk yield from each of the six herds.

## Introduction

Bulk milk tank somatic cell count (BMTSCC) is a long-standing marker of milk quality, impacting cheese production [1], milk flavor, and product shelf life [2]. Criteria for determining the permissible BMTSCC threshold vary among countries. A focus on mastitis—the major factor affecting BMTSCC—in herd management is crucial for both the farmer and the milk industry. SCC in the milk of uninfected mammary glands is low (10–100 × 10^3^ cells/mL) [3, 4], whereas intramammary infection is the single major cause of udder inflammation, which results in increased SCC and thus affects the quality and quantity of the produced milk [5, 6].

Milk from udders with clinical infection is not suitable for human consumption and is not added to the bulk milk tank, resulting in direct monetary losses for the farmer. BMTSCC is a measure of all of the healthy animals’ milk, including cows with lower milk quality and high SCC. In most industrial dairy operations, since the dairy cannot control every animal milked into the bulk tank, milk quality and price are regulated through the BMTSCC. Given that BMTSCC thresholds vary among countries, the question remains: to what level should it be decreased? High BMTSCC is an indicator of the overall udder health of the cows in the herd. To produce milk with low SCC, dairy farms have several options: control and/or treat mastitis and refrain from milking the glands/udder into the bulk milk tank, or cull the cow. Within this framework, every cow/day whose milk is not sent into the bulk tank decreases the farmer’s income, but its costs remain the same. For instance, if the infection requires treatment intervention such as antibiotics, this results in discarded milk for days; if the infected gland remains inflamed, it requires drying-off, or the cow must be culled. Moreover, because incentives for low BMTSCC and penalties for high BMTSCC determine milk prices, the farmer’s profits decrease even more. Farmers are faced with several dilemmas when a cow is suspected of having clinical, or even worse, subclinical chronic mastitis: among them is whether or not to treat the cow with antibiotics. Drawbacks include (i) type and sensitivity of the bacteria–additional cost for laboratory testing, veterinarian labor and drugs; (ii) discarding milk for days after treatment, regardless of recovery (loss of money); (iii) complying with strategies of antibiotic overuse due to the development of antimicrobial resistance over time, and therefore decrease of antibiotics use.

Cow-share-contribution to the BMTSCC refers to each cow’s milk volume sent to the bulk tank with respect to its relative addition to the bulk tank’s SCC. One unit of cow-share-contribution equals the addition of 1,000 cells to each mL of the bulk milk volume above the mean BMTSCC; e.g., if the mean BMTSCC is 200 × 10^3^ cells/mL and a cow with 10 units is removed from milking, the mean BMTSCC will be 190 ×10^3^ cells/mL. Thus, high-yielding cows with high SCC from mastitis are the main contributors to penalized milk prices, calling for a treatment designed to heal the infected gland/udder without the costs associated with routine mastitis treatments.

**Fig 1:**
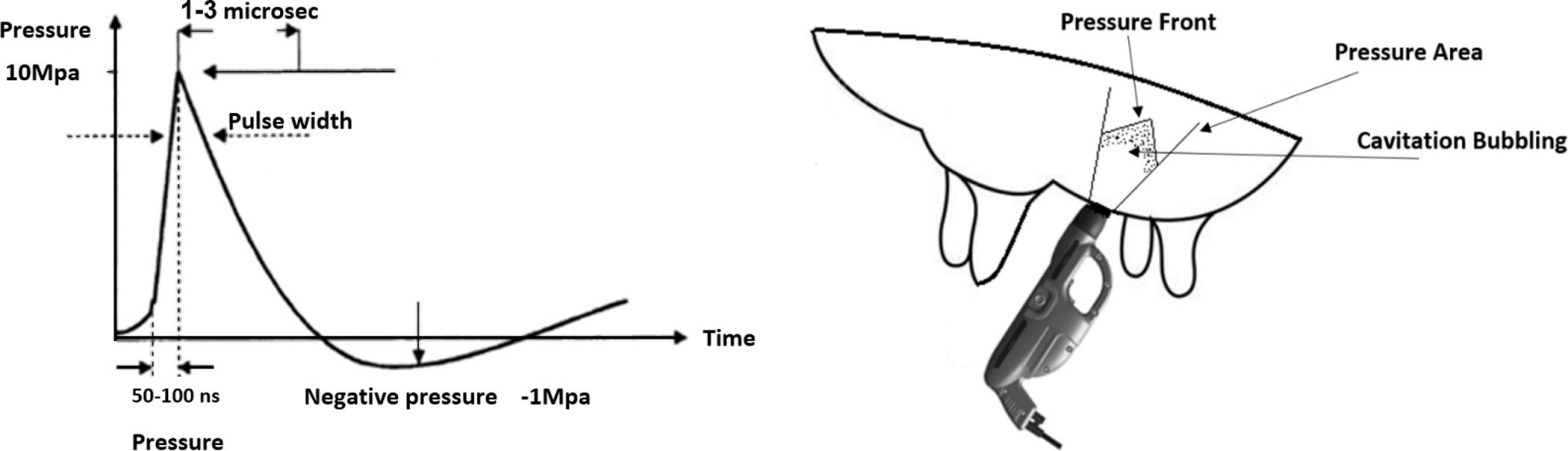
Acoustic pulse wave. An illustrated acoustic pulse wave (**1A**) and the positioning of the APT-X device on the cow’s udder (**1B**).

A recent option for treating mammary infections in dairy cows relies on the biological effect of low-intensity acoustic pulse technology (APT). This treatment affects cells by mechanotransduction, stimulating and remodeling the growth of new arterioles, and improving blood supply and oxygenation, all of which support faster healing and result in anti-inflammatory effects [7, 8] without the use of additional medication. APT is a short pulse of positive pressure (1–3 μs) followed by negative pressure (shear stress effect), causing microbubble and cavitation formation which disappear in a few hundred microseconds (Fig 1A) [9, 10]. The APT-X device developed by Armenta Ltd. (Ra’anana, Israel) was specifically adapted for the treatment of dairy cows (Fig 1B): it is harmless to the animal, it does not affect the milking routine, and no milk is discarded during its application [11]. Treatment of clinical and subclinical mastitis on commercial dairy farms using the APT-X has been shown to result in >70% recovery, reduce culling by >70%, and increase daily milk yield (MY) [11], and to give better results than treatment with antibiotics. The outcome of its use was increased income from milk and reduced losses from culling [12].

The aim of the present study was to assist in controlling BMTSCC by applying APT treatment after the monthly milk recording to every cow in the herd with a high share-contribution of SCC, and comparing the results to untreated control cows to evaluate its benefits.

## Materials and methods

### Study layout

A study was carried out dealing with high share-contribution of cells to the BMTSCC. Monthly milk recording data were reviewed using the Israeli Cattle Breeders Association (ICBA) database from April to August 2020, for six farms with 3,900 cows in total (Table 1). Approximately 300 cows were identified with >1.5 units of cow-share-contribution. Of those, the 206 top share-contributing cows were selected and divided into APT-treatment (n = 103) and untreated control (n = 103) groups. Selection and assignment for treatments was performed in each herd immediately after milk recording. Each pair of cows was selected according to lactation number, days in milk (DIM), daily milk yield (MY), SCC and SCC-share-contribution as appeared in the ICBA monthly report.

**Table 1:** The significance (*P* values) of the ANOVA results for the day effect and the R^2^.

Farm	Herd Size	APT	Control	Total
**1**	400	21	22	43
**2**	300	15	17	32
**3**	680	10	10	20
**4**	1140	26	21	47
**5**	1080	7	7	14
**6**	300	24	26	50
**Total**	3900	103	103	206

APT–acoustic pulse technology

At the time of the cows’ identification (t = 0) and assignment to the APT-treated or untreated control group, some of the cows had a first elevation of SCC (>800 × 10^3^ cells/mL), while the others had had high SCC (>800 × 10^3^ cells/mL) for 1–2 months before treatment; the latter were identified as having chronic inflammation. Each cow’s minimum contribution to the tank was 24 × 10^9^ cells (SCC × MY), for example, for a cow with SCC of 800,000 cells/mL and MY of 30 L. For 3 months post-treatment, milk recording was used to obtain data on MY and SCC and the relative share-contribution to the bulk tank for each herd on the day of milk recording. Because the data for identification were drawn from the monthly milk recording and time of treatment was 6–7 days, the following milk recording was 15, 45 and 75 d after the end of the treatment. Management decisions (drying off, culling) were at the farmer’s discretion; MY and share-contribution to the BMTSCC were calculated using ICBA data.

All methods were carried out in accordance with relevant guidelines and regulations and all treatment protocols were approved by the Institutional Animal Care Committee of the Agricultural Research Organization, the government-sanctioned body for such authorizations in Israel (IL 692/17).

### APT treatment protocol

An APT treatment session consisted of a total of 400 acoustic pulses at a frequency of 2.7 Hz (~2.5 min/treatment) and average acoustic energy density of 0.04 mJ/mm². The maximum penetration depth into the treated gland was 350 mm and the volume of tissue covered was 2.6×10^6^ mm^3^ (2.6 L). The acoustic pulses were delivered between the first and second daily milking. Full treatment included three sessions 3 days apart. At each session, 2 × 200 pulses were delivered over two regions around the teat (400 pulses per quarter), for a total of 1,200 pulses for the whole treatment cycle per treated quarter. The APT-X device was held in an upright position (Fig 1B), with the aim perpendicular to the surface of the ground.

### Statistical analysis

#### Recovery from the inflammation and individual cow-share-contribution

*Recovery*. Recovery from inflammation was determined as an >75% decrease in SCC in at least two of the three monthly milk recordings after treatment [13].

Differences between effect of treatments—APT or no treatment of cows with subclinical mastitis—on recovery: A multivariable model was designed with a logistic model statement using the GLIMMIX procedure of SAS (version 9.2, SAS Institute, Cary, NC, USA, 2009), with recovery as the dependent variable, as previously described [14]. The model was analyzed with the general form: Recovery rates = intercept + Herd + Group + Lactation number + DIM + error, where: Recovery rate = ln P/(1-P), P = probability of recovery; Herd = six different dairy farms; Group = cows treated with APT vs. untreated cows; Lactation number = first, second, third and more lactations days in milk. All variables were considered fixed effects except DIM, which was continuous.

*Contribution*. To calculate the changes in individual cows’ share-contribution, we used different equations relative to the changes in MY and share-contribution at each of the three monthly milk recordings after time of treatment. Monthly success of MY (MSM) was defined as changes in MY relative to time of treatment (t = 0) that were less than 10%, 15% and 20% in the first, second and third milk recording, respectively (t = 75 days). Monthly success of share-contribution (MSS) was defined as a decrease of >75% from day of treatment (t = 0) for all three monthly milk recordings. Finally, overall success was defined when two out of the three milk recordings were successful.

1. MSM = MY at first recording/t = 0 × 100 < 10%; second recording/t = 0 × 100 < 15%; third recording/t = 0 × 100 < 20%
2. Overall success = 2 out of 3 MSM
3. MSS = share-contribution at first, second or third recording/t = 0 ×100 > 75%
4. Overall success of share-contribution = 2 out of 3 MSS

Because the value of the cow-share-contribution is relative to herd size and BMTSCC on the day of the milk recording, first analyses of MSS in each of the six herds was conducted separately for each herd by χ^2^ test. We used the PROC MIXED procedure of SAS with the general form: SCC, MY or cow-share-contribution = intercept + Herd + Lactation number + Group + error, where Herd = six dairy farms; Lactation number = 1, 2, 3, or more lactations; Group = two experimental groups (APT vs. untreated) or subgroups (first elevation vs. chronic in the APT group). This analysis was conducted at each time point. Data are presented as means and SEM.

## Results

**Fig 2:**
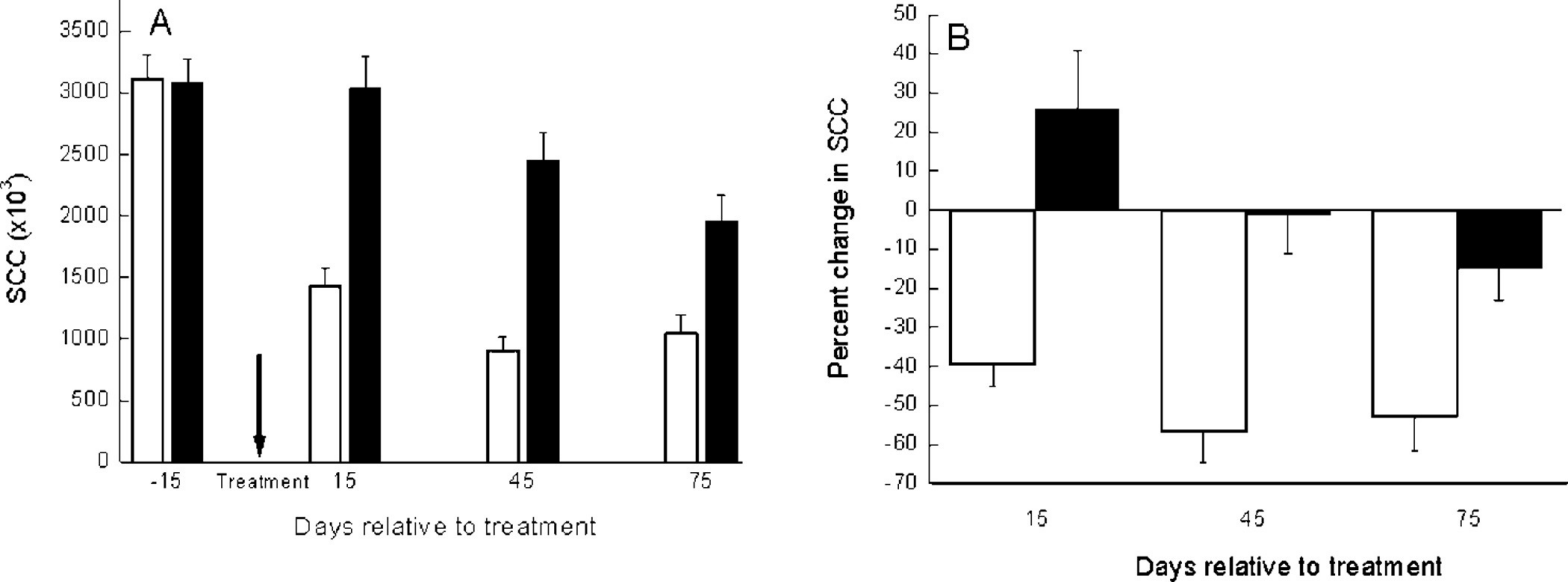
Mean and SE of somatic cell count. Mean and SE of somatic cell count (SCC × 10^3^) (**2A**) and the average percent individual change in SCC relative to time of treatment (t = 0) during the first 75 d post-treatment (**2B**) of 103 APT (□) and 103 untreated (■) cows with mastitis.

The distribution of the 206 cows according to farm and group is summarized in Table 1. During the 75 days of the study, of the 103 treated cows, 2 cows (1.9%) were culled, whereas of the 103 control cows, 19 (18.5%) were culled (*P* < 0.0001) and 5 (4.85%) infected quarters were dried-off. No difference was found between APT-treated and control groups in lactation number (2.75 ± 0.15 vs. 3.07 ± 0.16), SCC at time of treatment (3,115 ± 206 vs. 3,085 ± 198 (×10^3^ cells/mL), MY (40 ± 0.88 vs. 38 ± 0.87 L) and cow-share-contribution (5.97 ± 0.47 vs. 6.26 ± 0.39 units). The mean DIM for the APT group was fewer than that for the control group with a large SE (134 ± 7.8 vs. 163 ± 10.7 days). The mean SCC in the APT-treated group 15 days after treatment decreased to ~45% (3.11×10^6^ to 1.42×10^6^), whereas no change (3.08 × 10^6^ to 3.04 × 10^6^) was recorded in the untreated control group (Fig 2A). The change in the APT group was significant (*P* < 0.007). Moreover, for the APT group, the SCC contribution (in percent) to the tank per individual cow was reduced by 39.4%, 56.6% and 52.7% on day 15, 45 and 75, respectively. For the untreated group, individual cow’s contribution increased by 26% on day 15 and then decreased by 1% and then by 15% on days 45 and 75, respectively (Fig 2B). This last change was significant (*P* < 0.001). The reduction in the mean SCC of the untreated group was mainly due to culling cows and not to recovery from inflammation. Overall, recovery from inflammation, i.e., an >75% decrease in SCC in at least two of the three monthly milk recordings after treatment (75 days) was 57.3% in the APT-treated group and 14.6% in the untreated control group (*P* < 0.0001).

The change in MY, calculated as percent from treatment time of the cows that remained in the herds, was not significant. However, the total milk volume milked into the bulk tank was lower in the untreated control group due to the higher culling rate.

The change in MY, calculated as percent of treatment time of the cows that remained in the herds, was not significant. However, the total milk volume milked into the bulk tank was lower for the untreated control group due to the higher culling rate. Overall success of share-contribution in each of the six herds is summarized in Table 2. The difference between the APT and untreated groups was consistently positive for the APT group (Table 2).

**Table 2:** Percent success, defined as >75% reduction of cow-share-contribution in 2 out of 3 milk recordings following treatment at each of the 6 herds of the 206 cows treated by APT or untreated (as control).

Herd	APT (%)	Control (%)	Diff. of APT to Control	P [F]
**1**	60	0	60	0.013
**2**	65	38	27	0.060
**3**	45	0	45	0.004
**4**	50	24	26	NS
**5**	71	55	16	NS
**6**	65	15	50	0.001

APT–acoustic pulse technology

**Fig 3:**
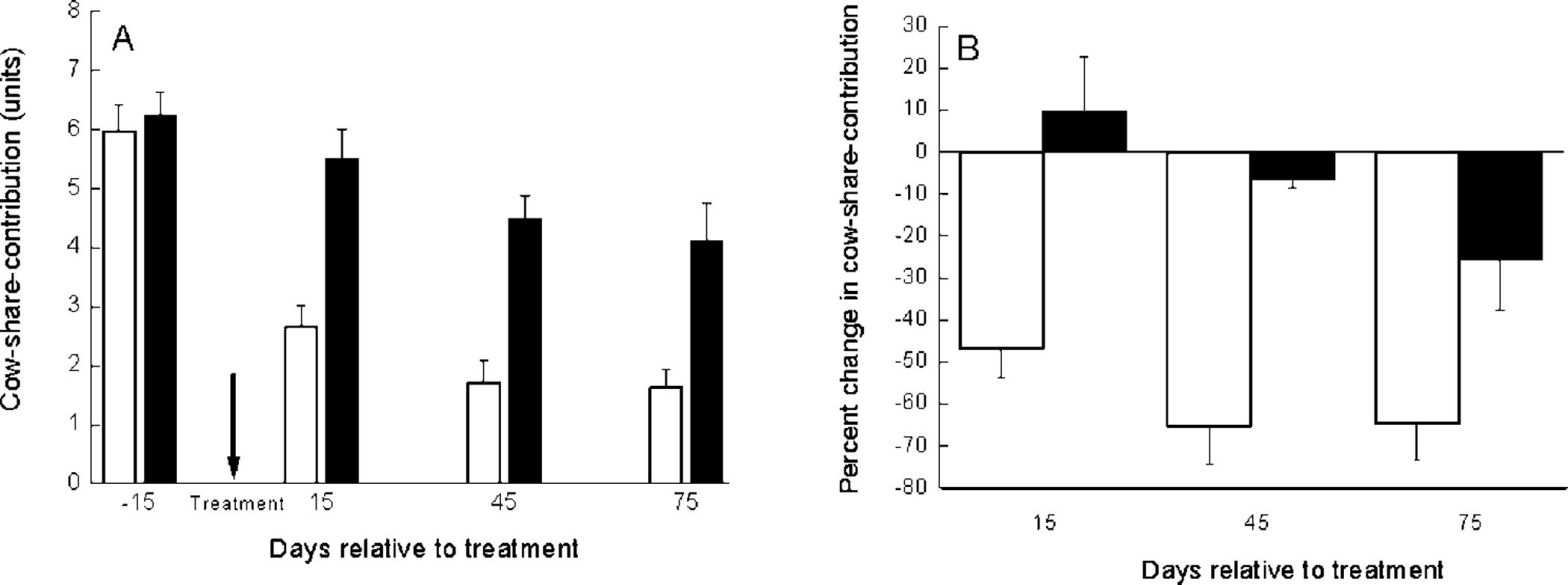
Cow-share-contribution of somatic cells to the bulk milk tank. Mean and SE of cow-share-contribution of somatic cells to the bulk milk tank (**3A**; arrow indicates time of treatment) and changes in individual cow-share-contribution (**3B**) relative to time of treatment (t = 0) during the first 75 d post-treatment of 103APT (□) and 103 untreated (■) cows with mastitis.

Although the value of cow-share-contribution is relative to herd size and BMTSCC, we further analyzed these arbitrary values in the six herds and included them in the statistical model. The mean values of cow-share-contribution above the BMTSCC were 5.97, 2.66, 1.72, and 1.63 for the APT group and 6.26, 5.52, 4.49, and 4.12 for the untreated control group on day 0, 15, 45, and 75, respectively (Fig 3A). This change was significant (*P* < 0.005). The mean cow-share-contribution at 15 days after treatment for the APT group decreased by 46.9%, whereas in the untreated control group, it increased by 9.7% (Fig 3B). The reduction became even more pronounced, up to ~65% in the APT-treated cows on days 45 and 75 vs. 6.6 and ~25.8% in the untreated group at the second and third milk recording, respectively (Fig 3B). This change was significant (*P* < 0.0001).

**Fig 4:**
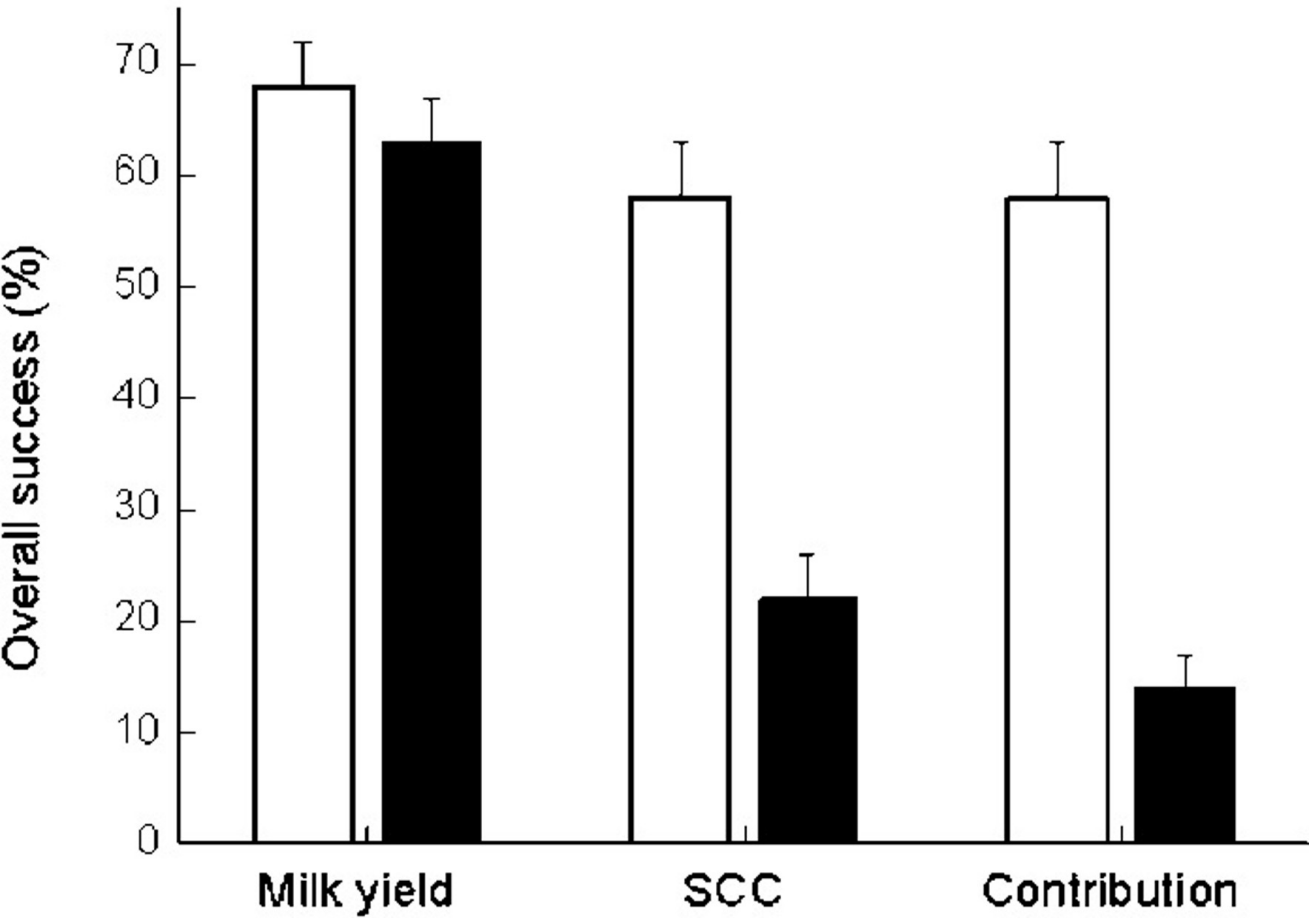
Percent success vs. milk yield, somatic cell count and cow-share-contribution. Percent of overall success of milk yield, somatic cell count (SCC) and cow-share-contribution of 103 APT (□) and 103 untreated (■) cows with mastitis.

Overall successes of MY, SCC and cow-share-contribution are illustrated in Fig 4. There was no difference in the overall success of MY between APT-treated cows and untreated controls, being ~65% for both. However, in the untreated control group, a high number of cows were culled and thus not included in this calculation. In contrast, the overall success of SCC reduction, and therefore of cow-share-contribution reduction, was significantly higher (*P* < 0.005) in the APT group than in the untreated controls, 58% vs. 22% for SCC and 56% vs. 14% for cow-share-contribution.

**Fig 5:**
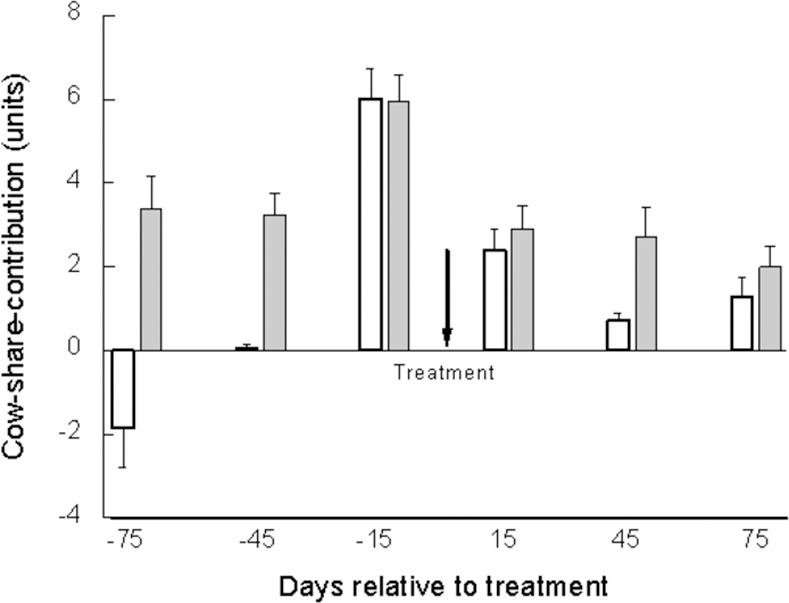
Cow-share-contribution of somatic cells to the bulk milk tank. Mean and SE of cow-share-contribution of somatic cells to the bulk milk tank relative to time of treatment (t = 0) during the first 75 d post-treatment of 103 APT separately for first elevation sub-group (□) (n = 64) and for the chronic sub-group cows (□) (n = 52). Arrow indicates time of treatment.

The next analysis was performed on the 103 APT-treated cows, separated into first-elevation (n = 51) and chronic (n = 52) subgroups. The reduction in SCC was more pronounced for the first-elevation vs. chronic cows treated by APT. Overall, recovery from inflammation, defined as an >75% decrease in SCC in at least two of the three monthly milk recordings after treatment, was significantly higher (*P* < 0.001) for the first-elevation subgroup (70%) than for the chronic cows (45%), whereas in the untreated control group (first elevation and chronic), only 15% recovered. Cow-share-contribution of somatic cells to the bulk milk tank decreased by 50% for the first-elevation subgroup and by 40% for the chronic subgroup at 15 days; the decrease continued (reaching 70–80%) in the first-elevation subgroup, but not in the chronic cows, which remained at the same level (Fig 5). Thus, significant differences were only found for days 45 and 75.

Based on this result, we performed a theoretical calculation of the impact of APT treatment on the high-share-contributing cows. Four assumptions were made for the simulation model: (i) all of the cows produce the same amount of milk, (ii) at any given time, 6% of the cows contribute a share of more than 1 unit to the mean BMTSCC (according to the ICBA milk recording), (iii) the total volume of milk in the bulk tank is 10,000 L, (iv) the remaining 94% of the cows contribute 100 × 10^3^ somatic cells/mL. According to this calculation, there was a decrease in on day 15 after treatment, and further decreases on days 45 and 75 (Table 3).

**Table 3:** Theoretical calculation of BMTSCC based on the results of the APT treatment impact of the higher share-contributing cows.

Cow-share-contribution		Bulk Milk	0	15	45	75
**% Under 1 unit**	94	9,400	100×10^3^	100×10^3^	100×10^3^	100×10^3^
**% Above 1unit**	6	600	3114×10^3^	1323×10^3^	837×10^3^	967×10^3^
**BMTSCC**			281×10^3^	173×10^3^	144×10^3^	145×10^3^

APT–acoustic pulse technology

BMTSCC–bulk milk tank somatic cell count

## Discussion

Introducing milk-quality parameters into the dairy industry highlights the need to prevent every cow/gland with low milk quality from being milked into the bulk tank. To eliminate milk from a mastitic cow, the most essential measurement is the SCC. Because the dairy cannot control every individual cow, bulk milk is graded and priced according to the BMTSCC. The regulatory limits for acceptable somatic cell level in milk at the dairy, and thus its quality grade, vary among countries. The outcome is that in all regulating countries, higher SCC leads to lower farm gate milk price. The major factor contributing to elevated BMTSCC is the highest share-contributing cows. These cows can be identified by testing individual cows with clinical mastitis and by routine milk recording for SCC. Management decisions about treatment—drying-off a quarter or culling the cow—are complex, as they depend on many factors, including the variety of treatment options. Thus, an assessment of highest share-contribution can serve as an additional tool in the farmer’s decision to control BMTSCC. We are aware that the cow-share-contribution value is relative to herd size and BMTSCC, i.e., a cow with high SCC in a large herd contributes less than a similar cow producing the same MY with the same SCC in a smaller herd. Therefore, in this study, we included a similar number of cows, with similar SCC and MY from each of the six herds.

Low-intensity shock waves affect cells by mechano-transduction currently applied to a wide range of pathologies of different localization in humans: wound healing and burns, scars [15, 16], bones and tendons [17–19], soft tissues and the local vasculature [20, 21]. The effect was also shown for ischemic heart regeneration [22] (more details can be found in the review of d’Agostino et al., 2015 [23]. Mechanotransduction is a biological pathway by which biomechanical forces are converted into biochemical responses, influencing some fundamental cell functions such as migration and proliferation [24]. The definition of Mechanotherapies is “promoting the homeostasis of healthy tissues by mechanical means at the molecular, cellular, or tissue level” [25].

Mastitis is an inflammation in the mammary gland mostly as a response to bacterial infection. During the infection, short and chronic disruption of mammary gland tissues by the immune cells progresses, which affects alveolar cuboidal epithelial structures and increase interlobular collagen rich areas with fibrous stroma and fat [26]. This process depends on the bacteria types and the time-span of the bacteria present in the gland. Moreover, in many infections, elimination of the bacteria is not enough to return to pre-inflammatory mode. This correlates with the chronic, long-term effects of mastitis caused by E. coli, indicating tissue damage and the process of cleaning damaged tissue, which results in decreased milk yield and reduced quality [27]. The APT is not aimed at killing the bacteria but rather indirectly reinforces the immune system that finally clears the bacteria. APT treatment transfers the mechanotransduction to the injured area, which stimulates arterioles remodeling and growth of new ones. The new blood vessels improve blood supply and oxygenation of the treated area and support faster healing together with the reducing of the inflammation, thus improving overall tissue functioning [7, 8] and resulting in higher milk yield and improved quality.

## Conclusions

The new APT treatment for clinical and subclinical mastitic cows with expected high share-contribution can be an appropriate treatment option for these cases due to: (i) high healing rate of the inflammation; (ii) its safety, with no systemic intervention, treating only the udder skin and therefore suitable for treating clinical and subclinical mastitis during lactation; (iii) significant reduction in the use of antibiotics thereby decreasing the need to discard milk during treatment; (iv) decreased culling of clinical and subclinical mastitic cows with high SCC. According to the results of the study, APT treatment of every cow with a high share-contribution to the BMTSCC could lead to its recovery, decreased SCC and improved MY. The difference in percent recovery between a new elevation and a chronic one suggests that by using APT treatment routinely, e.g., at every milk recording, most cows will be identified as having subclinical mastitis (new elevation) and thus the percentage of recovery will rise, significantly increasing the farmer’s economic gains: the overall quality of the bulk tank milk will improve, influencing the milk grading and increasing its price.

## Supporting information

S1 Dataset (XLSX)
